# Methylation status of individual CpG sites within *Alu *elements in the human genome and *Alu *hypomethylation in gastric carcinomas

**DOI:** 10.1186/1471-2407-10-44

**Published:** 2010-02-17

**Authors:** Shengyan Xiang, Zhaojun Liu, Baozhen Zhang, Jing Zhou, Bu-Dong Zhu, Jiafu Ji, Dajun Deng

**Affiliations:** 1 Key Laboratory of Carcinogenesis and Translational Research (Ministry of Education), Beijing Cancer Hospital and Institute, Peking University School of Oncology, Fu-Cheng-Lu, No.52, Haidian District, Beijing, 100142, China

## Abstract

**Background:**

*Alu *methylation is correlated with the overall level of DNA methylation and recombination activity of the genome. However, the maintenance and methylation status of each CpG site within *Alu *elements (*Alu*) and its methylation status have not well characterized. This information is useful for understanding natural status of *Alu *in the genome and helpful for developing an optimal assay to quantify *Alu *hypomethylation.

**Methods:**

Bisulfite clone sequencing was carried out in 14 human gastric samples initially. A *Cac*8I COBRA-DHPLC assay was developed to detect methylated-*Alu *proportion in cell lines and 48 paired gastric carcinomas and 55 gastritis samples. DHPLC data were statistically interpreted using SPSS version 16.0.

**Results:**

From the results of 427 *Alu *bisulfite clone sequences, we found that only 27.2% of CpG sites within *Alu *elements were preserved (4.6 of 17 analyzed CpGs, A ~ Q) and that 86.6% of remaining-CpGs were methylated. Deamination was the main reason for low preservation of methylation targets. A high correlation coefficient of methylation was observed between *Alu *clones and CpG site J (0.963), A (0.950), H (0.946), D (0.945). Comethylation of the sites H and J were used as an indicator of the proportion of methylated-*Alu *in a *Cac*8I COBRA-DHPLC assay. Validation studies showed that hypermethylation or hypomethylation of *Alu *elements in human cell lines could be detected sensitively by the assay after treatment with 5-aza-dC and M.*Sss*I, respectively. The proportion of methylated-*Alu *copies in gastric carcinomas (3.01%) was significantly lower than that in the corresponding normal samples (3.19%) and gastritis biopsies (3.23%).

**Conclusions:**

Most *Alu *CpG sites are deaminated in the genome. 27% of *Alu *CpG sites represented in our amplification products. 87% of the remaining CpG sites are methylated. *Alu *hypomethylation in primary gastric carcinomas could be detected with the *Cac*8I COBRA-DHPLC assay quantitatively.

## Background

The *Alu *element is a member of the SINE family of repetitive elements. It is an example of a non-automatic retrotransposon. It is the most abundant gene in the human genome (more than one million copies per haploid genome), representing 10% of the genome mass [1]. *Alu *elements are mainly distributed in gene-rich regions. About 75% of gene promoters in the genome contain *Alu *elements [2].

**Figure 1:**
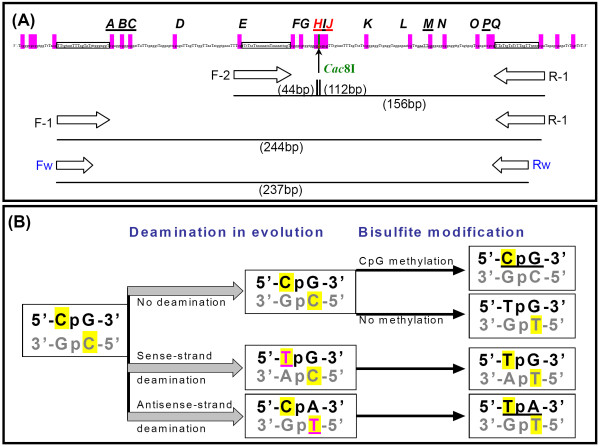
**Illustration of the genomic organization (A), methylation, and deamination (B) of CpG sites within *Alu***. The 17 CpG sites are marked with the capital letters (A-Q) above the *Alu *consensus sequence (Ref. [15]) and highlighted in the colour pink. The arrowed-line point to the recognition sequence (including the sites H and J) of restriction enzyme *Cac*8I; Rectangles, primer matching sequences; The arrowed-boxes, primer F-1, F-2, and R-1 were used to amplify the bisulfite-converted templates, and F-w and R-w were used to amplify the templates without bisulfite treatment. The primer F-2 and R-1 were the same as described (Ref. [14]). The sites **A-B-C **were included within the probe sequence of MethyLight (Ref. [15]). The sites **H-I-J **were the target CpGs in pyrosequencing assay and the site **P **was the target CpG in *Mbo*I-COBRA (Ref. [14]). The site **M **was the target CpG used for quantification of unmethylated *Alu *(Ref. [16]). The capital letter T in the colour pink was resulted from evolutionary deamination of cytosine. The underlined **CpG **and **TpA **represent the methylated CpG site and antisense-deaminated CpG site, respectively.

A consensus *Alu *element usually contains 24 CpG sites (Figure 1) [3]. In fact, the CpGs within *Alu *elements harbour up to one-third of the total CpG sites in the genome [4,5]. In normal tissues most *Alu *elements are methylated and transcriptionally inactive. However, stress-induced demethylation of these CpGs could reactivate *Alu *transcription. Although *Alu *transcripts encode no protein, they can regulate expression of other genes, affect recombination, and influence patterns of nucleosome formation and evolution of the genome [6-9]. Demethylation of *Alu *elements is an indicator of lower genome stability, which is necessary for gene recombination and chromosome translocation [10]. Retrotranscriptase encoded by *LINE-1 *helps retrotranscription and transposition of *Alu *elements into the genome [11]. Total methylation content of *Alu *elements and *LINE-1 *sequences is highly correlated with global DNA methylation content [12]. Estimation of total methylation content of *Alu *elements is useful for evaluation of the global genomic methylation status and level of homologous and non-homologous chromatin recombination in gene-rich regions.

Deamination and other variations of CpGs within *Alu *elements happen frequently during evolution. This has led to diversification of *Alu *into 213 subfamilies [13]. However, the maintenance and methylation status at each CpG site within *Alu *has not been characterized experimentally. Several methods such as the combined bisulfite restriction assay (COBRA), pyrosequencing, MethyLight and unmethylated *Alu*-specific amplification have been developed in the past few years [14-16]. In those assays, methylation content of a specific CpG site(s) within *Alu *elements was detected and used to represent the methylation/demethylation level of all *Alu *elements. However, representativity and consistency of methylation status of these used CpG sites have not been investigated. Additionally it is not known how representative or reproducible are the results for these previously studied CpG sites. In the present study, initially we analyzed the maintenance status and variations, including deamination, at each CpG site, in human cancer and normal tissues. Finally, the methylation status of each CpG site was then evaluated based upon extensive bisulfite clone sequencings. Based upon the analysis of the correlation between the number of methylated CpGs and the methylation status of each CpG site within *Alu *elements, a novel convenient COBRA-DHPLC assay was developed to quantify changes of the proportion of methylated-*Alu *elements in human gastric carcinomas successfully.

## Methods

### Human gastric mucosa samples

Forty-eight pairs of primary gastric carcinoma (GC) surgical tissues and their corresponding normal (GC-Nor) samples, 55 gastric biopsies from patients with or without gastritis, were collected from inpatients and outpatients at Beijing Cancer Hospital, respectively (male/female sex ratio, 7/3; 40-81 years old, the average age 60-y). All these specimens were freshly frozen at -70°C. The Institutional Review Boards of Peking University School of Oncology approved the study and all patients gave written informed consent.

### Cell lines and treatment of 5-aza-dC

Human carcinoma cell lines AGS and SW480 were cultured in F12 and DMEM medium (GIBCO) supplemented with 10% FBS at 37°C, 5% CO_2_. These cell lines were treated with 10 μmol/L (final concentration) of 5-aza-dC (Sigma) or an equal volume of PBS (pH7.4, 5 μl/well with 500 μl medium) for 72 hours, individually.

### DNA extraction and bisulfite modification

Genomic DNA was extracted from tissue samples with phenol/chloroform as described [17]. The unmethylated-cytosines of the genomic DNA were converted to uridines by addition of 5 mol/L of sodium bisulfite at 50°C overnight [18].

### Modification with M.*Sss*I

Genomic DNA (0.1 μg/μl) of AGS and SW480 cell lines was treated with M.*Sss*I methylase (final concentration, 1 U/μg DNA; New England Biolabs) and 0.16 mmol/L of *S*-adenosylmethionine (SAM) at 37°C overnight.

### Amplification of *Alu *elements by PCR

Three sets of primers were used and illustrated in Figure 1A. The primer sequences of R-1 and F-2 were the same as described [14]. Other primers were designed according to the *Alu *consensus sequence [15]. The universal primer set-1 (F-1: 5'-TTtg taatT TTagT aTttt gggag gT-3'; R-1: 5'-gatcc ccaAA ctAAaA tAcaA tAA-3') was used to amplify a 224-bp *Alu *fragment (including 17 CpG sites from site A-Q) using the bisulfite-modified templates by PCR-1 [30 μl reaction including 50 ng DNA templates, 0.15 mmol/L of dNTP, 0.15 μmol/L of each primer, 0.9 U of HotStar *Taq *DNA polymerase (Qiagen GmbH, Hilden, Germany); thermal cycler conditions: 95°C for 15 min → (95°C for 30 s → 60°C for 30 s → 72°C for 30 s) × 37 cycles → 72°C for 10 min]. The universal primer set-2, consisting of the primer F-2 (5'-gatct TtTta Ttaaa aataT aaaaa ttagT-3') and the above primer R-1 was used to amplify a 156-bp fragment (including 12 CpG sites: Site F-Q) with the bisulfite-modified templates by PCR-2 [thermal cycle condition: 95°C for 15 min → (95°C for 30 s → 52.8°C for 30 s → 72°C for 30 s) × 37 cycles → 72°C for 10 min] as described [14]. Both the primer set-1 and set-2 contain a number of T (or A) in the forward primers (or reversed primer), with which only bisulfite-modified templates could be amplified. The primer set-w (Fw: 5'-gcctg taatc ccagc act-3'; Rw: 5'-aggct ggagt gcagt gg-3') was used to amplify a 237-bp *Alu *fragment from genomic DNA without bisulfite modification by PCR-W. The reaction mixture was the same as the PCR-1 [thermal cycler conditions: 95°C for 15 min → (95°C for 30 sec → 60°C for 30 sec → 72°C for 30 sec) × 37 cycles → 72°C for 10 min].

### Cloning and sequencing

Above PCR-1, PCR-2 and PCR-w products were cloned into TA-vector and sequenced by ABI 3730 Analyzer. The average number of the sequenced TA clones was 30 for each sample.

### *Cac*8I CORBA of the PCR-2 products

After bisulfite conversion, the 156-bp PCR-2 products of methylated *Alu *templates contain a 5'-G**Cg**nG**Cg**-3'sequence (including CpG sites H and J), that can be digested by *Cac*8I (Figure 1A). Thus, *Cac*8I digestion was used to develop the COBRA assay for detection of *Alu *methylation. The PCR-2 products (10 μL) were digested with 2 U of *Cac*8I (New England Biolabs) at 37°C for 6 hours. The 156-bp methylated *Alu *was cut into 112-bp and 44-bp fragments by *Cac*8I, whereas the unmethylated *Alu *was not cut because these cleavage positions did not exist after bisulfite modification. The digested PCR products were further analyzed directly by DHPLC without purification. Equal amount of PCR-2 products of a fully methylated *Alu *clone was used as the standard *Cac*8I digestion control in every COBRA experiments. Fully cut of all of the standard control products was used as the indicator of the complete digestion of tested samples.

### Separation and quantification of methylated- and unmethylated-*Alu *by DHPLC

The *Cac*8I cut methylated-*Alu *and uncut unmethylated-*Alu *fragments in the PCR-2 digestion were separated with the WAVE DNA Fragment Analysis System (Transgenomic, Inc., Omaha, USA) at 48°C, the non-denaturing temperature optimised for analysis of double-stranded DNA fragments amplified from bisulfite-modified templates as described previously [19,20], and detected by fluorescence (FL)-detector [21]. The WAVE-HS1 FL-dye buffer (Transgenomic, Inc.) was used to enhance the FL-intensity of PCR products (universal post-column labelling). The apparent total methylated-*Alu *proportion in tested samples was calculated according to the ratio of the peak height for the 112-bp methylated fragment to the total peak height of both the methylated and 156-bp unmethylated fragment peaks. The peak height of the unmethylated-*Alu *PCR products was 81.22% of that for the *Cac*8I digested methylated-*Alu *PCR products at equal molecule number. Therefore 81.22% was the constant used to adjust the measured peak height for the methylated-*Alu*.

### Data and statistical analysis

The Student's t-test was used to analyze the average methylation frequency of each CpG site within the sequenced clones and the total methylated-*Alu *proportion in different groups of gastric mucosa samples. The SPSS 16.0 software was used for these statistical analyses. Significance was defined as *p *< 0.05. The correlation coefficient between methylation of each CpG site and the methylated-*Alu *was calculated.

## Results and Discussions

### Analysis of methylation and mutation status of each CpG site within *Alu *elements in human gastric samples by bisulfite clone sequencing

**Figure 2:**
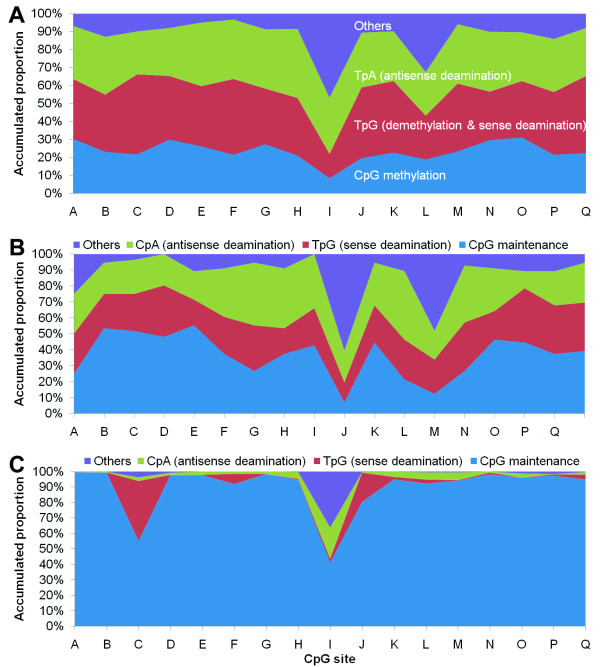
**Proportion of methylation and variations at each CpG site within *Alu *elements**. The average proportion of methylation (CpG), deamination of CpG on the antisense strand (TpA), demethylation and deamination of CpG on the sense strand (TpG), and other variations at each CpG site among a total of 427 bisulfite clones from 14 tested gastric samples is illustrated (A). Maintenance, deamination on the sense strand (TpG) and antisense strand (CpA) at each CpG site were analyzed based on 56 tested clone sequences from a gastric mucosa sample without bisulfite modification (B) and on 476,152 copies of Alu elements extracted from the NCBI database of the human genome (C) as described bioinformatically (Ref. [13]).

The methylation status of each CpG site within the full-length *Alu *elements in both cancer and normal tissues has not previously been reported. Therefore, we carried out extensive bisulfite clone sequencing of the 224-bp PCR-1 products amplified with the primer set-1 (Figure 1A). The PCR-1 products, including 17 CpG sites (A ~ Q), were amplified from bisulfite-modified templates of 14 gastric mucosa samples [including 5 pairs of GCs and GC-Nor tissues, and 4 normal gastric mucosa biopsies], cloned, and sequenced (about 30 clones per sample). Results showed that the methylation frequencies were quite variable among different CpG sites (7.1% ~ 33.9%). Compared with the GC-Nor samples, hypomethylation in GC samples was not observed among all of 17 CpG sites when sequencing. The average methylation rate of these 17 CpG sites was 22.7% in normal biopsy control, 23.3% in GC-Nor, 24.6% in GC (Table 1). Both the lowest methylation rate and highest positive rates of other kinds of mutations were observed at the sites I and L (Table 1 and Figure 2A). A significant difference of CpG retention rate was observed at the site L between GC-Nor and normal control samples (*P *= 0.022).

**Table 1:** Comparison of positive rates of CpG, TpG, TpA (deamination on the antisense strand), and other kinds of mutations at each CpG site between 4 normal gastric biopsies (Normal) and 5 pairs of gastric carcinoma tissues (GC) and the corresponding normal tissues (GC-Nor) (mean ± SD, %)

CpG site	CpG			TpG			TpA^a^			Others		
	
	Normal	GC-Nor	GC	Normal	GC-Nor	GC	Normal	GC-Nor	GC	Normal	GC-Nor	GC
A	32.8 ± 9.9	28.7 ± 8.0	29.8 ± 8.8	32.6 ± 9.0	32.4 ± 7.7	33.5 ± 6.4	27.7 ± 2.7	30.1 ± 6.2	30.7 ± 6.3	7.0 ± 7.0	8.7 ± 5.9	6.0 ± 4.0
**B**	22.0 ± 3.3	23.7 ± 6.8	24.5 ± 10.1	36.1 ± 5.1	30.8 ± 18.1	28.0 ± 6.1	29.3 ± 3.8	32.2 ± 7.3	35.4 ± 6.1	12.6 ± 1.4	13.4 ± 6.4	12.1 ± 8.6
**C**	22.5 ± 9.2	21.2 ± 4.0	22.0 ± 11.6	44.3 ± 7.8	43.2 ± 7.4	46.8 ± 7.6	19.7 ± 14.2	28.6 ± 9.3	22.2 ± 9.0	**13.5 ± 2.9**	**7.1 ± 2.3^b^**	8.9 ± 5.5
**D**	25.4 ± 12.3	29.7 ± 8.6	30.3 ± 10.8	34.8 ± 11.1	29.0 ± 6.0	42.6 ± 15.1	31.1 ± 13.0	26.0 ± 8.6	22.9 ± 6.1	8.7 ± 2.2	**15.3 ± 6.3**	**4.1 ± 2.6^c^**
**E**	19.9 ± 9.6	30.2 ± 6.6	27.4 ± 6.6	35.1 ± 9.6	31.0 ± +7.4	34.9 ± 5.0	42.0 ± 11.3	34.3 ± 6.2	30.5 ± 4.7	3.0 ± 2.4	4.5 ± 4.0	7.3 ± 3.2
**F**	17.9 ± 4.7	24.9 ± 7.3	21.6 ± 5.4	38.7 ± 0.2	39.2 ± 9.3	48.4 ± 8.0	36.9 ± 1.4	33.8 ± 8.1	27.2 ± 5.7	6.5 ± 4.6	2.0 ± 1.5	2.8 ± 1.7
**G**	24.3 ± 7.4	24.2 ± 4.1	33.9 ± 5.8	30.5 ± 8.1	31.7 ± 4.6	29.4 ± 5.0	38.5 ± 2.2	33.8 ± 10.6	27.9 ± 6.1	6.7 ± 6.4	10.3 ± 6.7	8.7 ± 5.7
H	22.1 ± 13.4	20.0 ± 6.5	21.8 ± 5.3	32.3 ± 14.5	32.7 ± 12.8	32.1 ± 6.8	34.2 ± 11.1	39.3 ± 8.3	39.4 ± 11.8	11.5 ± 8.2	7.9 ± 3.2	6.7 ± 3.0
**I**	9.4 ± 5.2	7.1 ± 4.7	8.6 ± 4.6	11.3 ± 5.5	15.4 ± 7.3	13.7 ± 3.6	36.1 ± 11.1	31.2 ± 9.9	27.7 ± 10.0	43.1 ± 14.6	46.2 ± 6.9	49.9 ± 7.1
**J**	18.3 ± 8.2	20.6 ± 3.0	20.2 ± 12.6	39.9 ± 5.9	38.3 ± 6.8	41.3 ± 15.7	32.6 ± 7.8	28.8 ± 5.7	30.1 ± 8.7	9.1 ± 5.9	12.3 ± 5.0	8.4 ± 4.7
**K**	22.9 ± 10.0	23.3 ± 2.8	21.6 ± 7.0	39.5 ± 7.1	43.2 ± 4.1	37.2 ± 12.5	24.2 ± 7.5	24.2 ± 5.6	30.8 ± 14.9	13.4 ± 6.5	9.3 ± 3.7	10.3 ± 2.4
**L**	**22.8 ± 1.4**	**14.8 ± 4.6^d^**	20.0 ± 9.4	24.1 ± 12.6	27.9 ± 15.0	20.7 ± 2.1	26.9 ± 13.2	19.4 ± 11.8	25.4 ± 4.4	26.2 ± 6.4	37.9 ± 27.6	33.9 ± 8.0
**M**	27.4 ± 7.3	21.6 ± 8.4	22.8 ± 4.6	39.6 ± 7.0	36.4 ± 7.7	35.5 ± 12.7	30.8 ± 4.0	34.7 ± 8.4	34.4 ± 7.0	2.2 ± 5.0	7.4 ± 5.8	7.3 ± 5.5
**N**	27.7 ± 4.3	27.2 ± 9.5	35.3 ± 7.7	31.4 ± 4.0	27.2 ± 7.6	22.3 ± 8.2	35.0 ± 8.9	33.4 ± 9.9	30.9 ± 4.9	5.9 ± 7.1	12.2 ± 6.4	11.5 ± 2.7
**O**	28.4 ± 7.8	32.2 ± 9.2	32.9 ± 8.8	37.2 ± 11.9	23.4 ± 7.0	33.6 ± 6.5	**22.6 ± 11.5**	**34.4 ± 10.6^e^**	24.3 ± 3.4	11.8 ± 5.5	9.9 ± 6.1	9.3 ± 4.7
**P**	20.1 ± 6.5	21.3 ± 4.2	22.7 ± 7.3	32.5 ± 16.0	35.7 ± 3.6	37.0 ± 11.7	33.1 ± 12.7	27.8 ± 9.8	27.0 ± 5.8	14.3 ± 6.4	15.2 ± 10.3	13.3 ± 5.2
**Q**	21.8 ± 8.9	24.8 ± 10.5	21.9 ± 6.9	40.8 ± 4.7	41.5 ± 7.6	44.6 ± 9.2	27.8 ± 6.0	26.2 ± 6.7	26.9 ± 10.7	9.6 ± 5.8	7.4 ± 4.6	6.6 ± 3.2

**Mean**	22.7 ± 7.6	23.3 ± 6.4	24.6 ± 7.8	34.2 ± 7.6	32.9 ± 7.3	34.2 ± 9.4	31.1 ± 5.9	30.5+4.8	29.0 ± 4.5	12.1 ± 9.7	13.4 ± 11.5	12.2 ± 11.9

**(Total)**	(23.6 ± 7.6)			(33.8 ± 8.0)			(30.2 ± 5.1)			(12.5 ± 10.8)		

^a ^representation of deamination on the antisense strand; ^b ^Normal vs. GC-Nor, *p *= 0.022; ^c ^GC vs. GC-Nor, *p *= 0.015; ^d/e ^Normal vs. GC-Nor, *p *= 0.022/0.046

Cytosine deamination at CpG sites, especially at the methylated-CpG sites, is a frequent event during evolution of *Alu *elements. The deamination on the antisense strand (antisense-deamination, CpA) was represented as TpA in the bisulfite-modified sequences specifically (Figure 1B). Significant difference in the antisense-deamination rate was found only at the site O when comparing the GC-Nor and normal samples (34.4% vs. 22.6%, *p *= 0.046), but not at any site when comparing the GC and GC-Nor (Table 1). These results suggest that the total antisense-deamination level in *Alu *elements was not significantly changed during carcinogenesis. Therefore, the results of total 427 sequenced clones were pooled together for further analysis.

Price et al. extracted about 480,000 *Alu *elements via a BLAST search of the human genome and sub-classified them into 213 subfamilies [13]. Using the same data, we did a bioinformatic analysis of the maintenance and variation status at each CpG site within *Alu *elements. According to this analysis, we found that the average rate of retention of the 17 CpG sites within *Alu *was up to 89.7% and that average rates of antisense-deamination (CpA), sense-deamination (TpG) and other variations were only 3.1%, 4.6%, and 2.7%, respectively (Figure 2C). However, in the present study, the average antisense-deamination rate was up to 30.2% among the 472 bisulfite-clones (Table 1). To study whether the high deamination rate in the tested samples was resulted from bisulfite modification bias, we carried out clone sequencing of *Alu *elements in one representative pair of GC and GC-Nor samples without bisulfite modification with the primer set-w (Figure 1A and Figure 2B). The results showed the average rate of deamination between the GC (27 clones) and GC-Nor (29 clones) samples was similar: 24.8% and 27.6% for the antisense strand, and 23.5% and 24.5% for the sense strand in the GC and GC-Nor sample, respectively. These results were consistent with 30.2% antisense-deamination rate among the 427 clones. Thus, bisulfite modification bias is unlikely the reason of high deamination rate in the genome observed in the present study.

Yang et al. also reported that based upon sequence analysis of 15 bisulfite clones almost two-thirds of the CpG sites in *Alu *elements are mutated [14]. The primer set-1 used in the bisulfite clone sequencing covers 470,988 of the extracted 476,152 *Alu *elements (98.9%). Among the 427 *Alu *clones, 69.3%, 18.5%, and 12.2% are *AluS*, *AluY*, and *AluJ*, respectively (Table 2). These results are similar to the values observed both in the extracted *Alu *database in the genome (73.7%, 20.1%, and 6.2%) and in the primer set-1 matched *Alu *subfamilies in the database (73.6%, 19.3%, and 6.1%). Moreover, among the 222 methylated *Alu *clones (with number of methylated-CpGs ≥ 4), proportions of methylated *AluS*, *AluY*, and *AluJ *are 70.3%, 23.4%, and 6.3%, respectively (Table 2). Thus, the possible PCR-1 bias, if any, may not result in favouring amplification of certain kinds of *Alu *subfamilies, especially for the methylated *Alu *elements. Because methylation-independent CpG-free primers are favouring the amplification of unmethylated (and evolutionary deaminated) sequences generally, we could not exclude that PCR bias for GC-poor *Alu *elements might lead to the result of low prevalence of CpG sites within *Alu *elements in the present study. The bias is likely unavoidable during amplification of the diversified *Alu *elements with PCR.

**Table 2:** Bioinformatic analysis of Content and distribution of *Alu *subfamilies among the PCR-1 and PCR-2 products Table legend text.

*Alu *subfamily	Bioinformatic analysis with the database^a^			Analysis of bisulfite clone sequencings		
	
	Average CpG no.^b^	Copy no. of *Alu*	Copy no. of *Alu *matched to the primers for PCR-1	Clone no. of *Alu *obtained in the PCR-1 products	Total clone no. of *Alu *with methylated-CpG number ≥ 4 in the PCR-1 products	Clone no. of *Alu *obtained in the PCR-2 products^c^
**AluJ**	16.3	29508 (6.2%)	28889 (6.1%)	52 (12.2%)	14 (6.3%)	3 (5.6%)
**AluS**	22.4	350888 (73.7%)	350419 (73.6%)	296 (69.3%)	156 (70.3%)	30 (55.6%)
**AluY**	24.5	95756 (20.1%)	91680 (19.3%)	79 (18.5%)	52 (23.4%)	21 (38.9%)

**(Total)**	22.4	476152 (100%)	470988 (100%)	427 (100%)	222 (100%)	54 (100%)

^a^476152 copies of *Alu *elements extracted bioinformatically from the NCBI database of human genome (C) as described (Ref. [13]);

^b ^within the full sequence of *Alu *elements as illustrated in Figure 1A;

^c^amplified from two representative samples with the primer set-2 after bisulfite modification

### Selection of methylated-CpG sites correlated well with methylated-*Alu*

Based on the sequencing results of the above 427 clones, we found that the average frequency of methylated CpG at each CpG site (based on the consensus *Alu *sequence) was 23.6%; TpG sites, 33.8%; TpA sites, 30.2%; other kinds of mutations, 12.5% (Table 1). TpG sites represent both the unmethylated CpGs modified with bisulfite and the evolutionary sense-deaminated CpGs. In the case of the average frequency of sense-deamination equal to that of antisense-deamination, as demonstrated with sequencing of the PCR-w products, the frequency of unmethylated CpG on each CpG site was 3.6% (the difference of 33.8% and 30.2%). It means that only 27.2% [the sum of 23.6% and 3.6%] of *Alu *CpG sites is retained (4.62 CpG sites/clone) and that 86.6% (23.6% of 27.2%) of the remaining methylation target-CpG sites within *Alu *elements are methylated in the genome.

**Figure 3:**
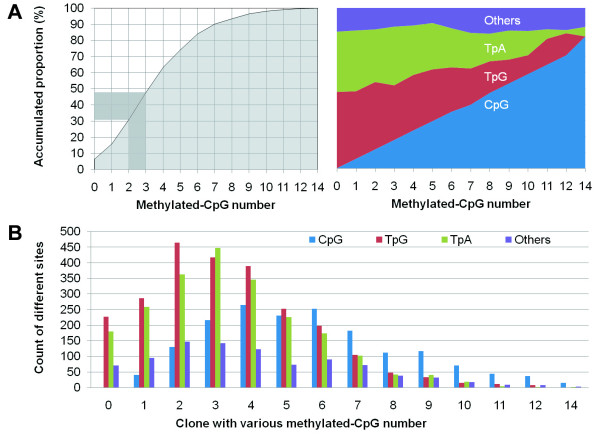
**Distribution of bisulfite *Alu *clones with different methylated-CpG number and variations at CpG sites**. The accumulated proportion of bisulfite clones with different number of methylated-CpGs (A, left), and the constituent ratio of various dinucleotides at the 17 CpG sites of *Alu *clones (A, right). The distribution of the number of CpG, TpG, TpA, and other variations among *Alu *clones from bisulfite-treated templates with a different number of methylated-CpGs was illustrated (B).

We further analyzed the distribution of frequencies of clones with different numbers of methylated-CpG sites; and found that 31% of clones (n = 133) contained 0~2 methylated CpG sites and 52% of clones (n = 222) contained 4~14 methylated CpG sites (Figure 3A, left). Based on the phenomenon that the number of methylated CpG sites in clones is negatively correlated with the number of both TpG and TpA in the same clones, respectively (Figure 3A right, and 3B), we conclude that it is the deamination on the sense and antisense strands, but not demethylation nor unmethylation, is the contributory factor to the low number of methylated CpG sites within these clones.

For localization of the CpG sites with good representation of methylation of *Alu *elements, the correlation of methylation status between each CpG site and the completely tested fragment of *Alu *was then calculated. When the clones contain 0-2 and ≥ 4 methylated-CpG sites were defined as the unmethylated and methylated *Alu*, respectively, the top four correlation coefficients were 0.963 for the site J, 0.950 for the site A, 0.946 for the site H, and 0.945 for the site D. The bottom four were 0.072 for the site I, 0.446 for the site L, 0.677 for the site O, and 0.692 for the site P (Additional file 1, Table S1). Apparently, the site P, which was used as the restriction site in the *Mbo*I COBRA assay [14], may not be a good detecting target. The combined coefficient was 0.849 for the sites A-B-C, which was used as MethyLight probe sequence [15], and 0.708 for the sites H and J, which was used in pyrosequencing [14].

### Development of a COBRA-DHPLC assay to quantify the proportion of methylated-*Alu *copies

Although the combined coefficients for the sites A-B-C and H&J were lower than their individual coefficients (Additional file 1, Table S1), it is reasonable to expect that comethylation of these CpG sites might have a higher representativity for methylated-*Alu *than for a single CpG methylation. In fact, the specificity of detection of the proportion of methylated-*Alu *by comethylation of the sites A-B-C or H&J was up to 100%, whereas for individual CpG methylation it was lower: 97.0% for the site J, 95.5% for the site H, 93.2% for the site A, and 90.2% for the site D, if the *Alu *clones contained 0~2 methylated CpG sites were considered as unmethylated-*Alu*. Thus, we used the strategy of detection of comethylation of the sites H&J to develop the following quantitative assay.

As mentioned above, pyrosequencing was previously used to detect *Alu *methylation at H-I-J sites [14]. However, unlike molecule-based multiple CpG sites-assays (i.e. clones or PCR copies) such as methylation-specific PCR, MethyLight, DHPLC, and clone sequencing, pyrosequencing is not a molecule-based assay, as it only provides information on the proportion of methylation at individually tested CpG site in the pooled *Alu *elements. The pyrosequencing results for different CpG sites might represent different *Alu *copies, respectively, thus should not be considered as a molecule-based assay. COBRA is one of the most convenient methods for detection of DNA methylation. When more than one CpG site is included in the restriction sequence, COBRA is also a molecule-based multiple CpG sites-assay, which could be used to detect methylated *Alu *copies among the genome.

**Figure 4:**
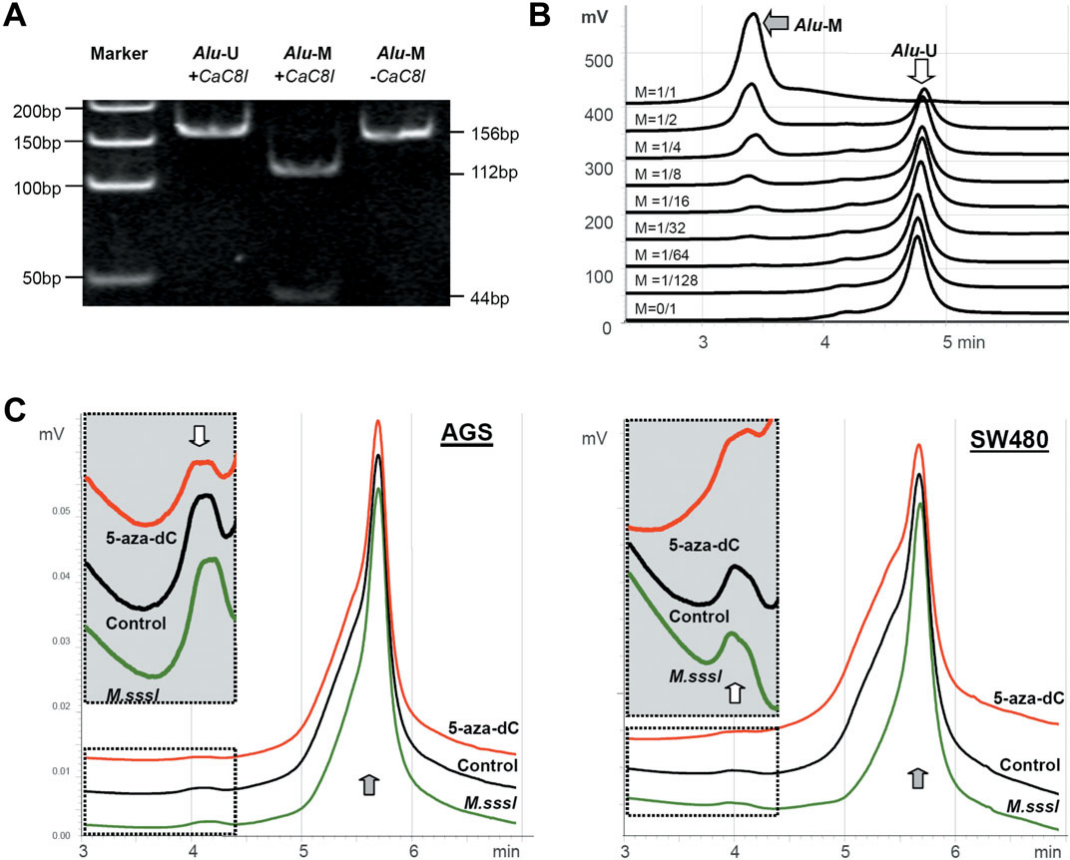
**Chromatography of *Cac*8I digestion of methylated and unmethylated PCR-2 products**. The electrophoresis image of the PCR-2 products of methylated- and unmethylated-*Alu *clones with and without *Cac*8I digestion (A); DHPLC chromatography of *Cac*8I digested products after the methylated-*Alu *PCR-2 products was diluted with the unmethylated *Alu *PCR-2 products at various ratios (B); After 5-aza-dC treatment (10 μM) or M.*Sss*I-modification, changes of the methylated-*Alu *proportion could be detected by the *Cac*8I COBRA-DHPLC assay sensitively (C). Open arrow and gray arrow point to peaks that correspond to the methylated- and unmethylated-Alu fragments, respectively. The gray square area is the single direction magnified part of open dash-line enclosed area.

To develop a COBRA assay suitable for various kinds of sample storages, such as paraffin embedded tissues, the optimal size of PCR amplicon should be less than 200-bp and a single restriction site should be selected for analysis. However, we could not find a restriction enzyme with a single cut site for the sites A, D, H, and J within the PCR-1 products. Therefore, the primer set-2 was used to amplify the 156-bp PCR-2 product that contains both H and J sites and could be digested by *Cac*8I (recognition site, 5'-GCN^NGC-3') when the template is methylated (Figure 1A). The PCR-2 products comethylated at both the sites H and J contain a 5'-GCg^nGCg-3' sequence, thus can be digested into 112-bp and 44-bp fragments by *Cac*8I (Figure 1A and 4A). Results of clone sequencing of the PCR-2 products from two representative samples showed that the primer set-2 was likely favouring the amplification of *AluY *clones (38%), which remain more methylation targets than *AluS *and *AluJ *(Table 2).

The traditional COBRA assay is gel-based and could only be used to detect target CpG methylation hemi-quantitatively. DHPLC is a typical separation and quantification method that could also be used to detect DNA fragments and methylation of CpG islands, whether or not it is combined with other assays (19,20,22). To detect the methylated-*Alu *proportion accurately, DHPLC was used to separate and quantify the *Cac*8I-cut (112-bp, methylated) and -uncut (156-bp, unmethylated) fragments. The methylated- and unmethylated-*Alu *in the digestion could be separated by DHPLC under the completely non-denaturing temperature 48°C. The retention time for the methylated- and unmethylated-*Alu *peak was 3.3 and 4.7 min, respectively. A linear relationship over a wide range could be observed between the loading concentration (1/1~1/64) and ratio of peak height for the methylated-*Alu *products (y = 0.9912x, R^2 ^= 0.995) (Figure 4B). The detection limit of this assay was about 3.4 × 10^6 ^copies of methylated *Alu *elements (the total copy number of *Alu *within one diploid cell is 2 × 10^6^). The coefficient of variation (CV) of this assay was 7.4%. Because of the very high specificity (100%) of comethylation at both the sites H and J for the methylated *Alu*, we used ratio of the 112-bp methylated *Alu *peak to the sum of the methylated and 156-bp unmethylated *Alu *peaks to represent the proportion of the methylated *Alu *copies in the tested samples, as described on the method section.

**Figure 5:**
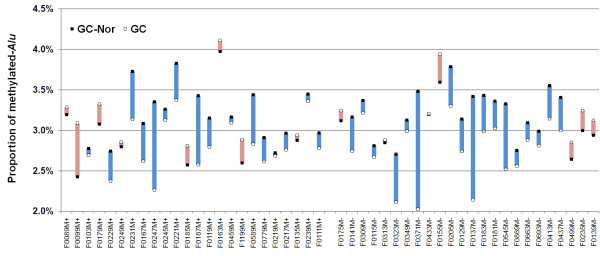
**Comparison of proportion of methylated-*Alu *in primary gastric carcinomas and the corresponding normal samples by the *Cac*8I COBRA-DHPLC assay**. Bisulfite modified *Alu *elements in 48 pairs of gastric carcinomas (GC) and the corresponding normal tissues (GC-Nor) with or without lymph node metastasis (M+/M-) were amplified with the primer set-2. The 156-bp PCR-2 products of *Alu *elements were digested with *Cac*8I at 37°C for 6 hours. The methylated-*Alu *was cut and the unmethylated-*Alu *was not cut by *Cac*8I. The digested PCR-2 products were separated by DHPLC at the undenatured temperature 48°C. The proportion of methylated-*Alu *was calculated according to ratio of the adjusted peak height for the methylated-*Alu *to that for the unmethylated-*Alu*. Hypomethylation was observed in 33 of 48 of GCs and marked with the colour blue.

Both hypomethylation of *Alu *elements in 5-aza-dC treated AGS (2.12% → 1.90%) or SW480 cell lines (2.28% → 1.88%) and hypermethylation of *Alu *elements in M.*Sss*I-methylated DNA templates (2.26% → 2.44% for AGS; 2.21% → 2.55% for SW480) could sensitively be detected by the *Cac*8I COBRA-DHPLC assay successfully (Figure 4C). It was reported that global genomic 5-methylcytosine content in the human genome was tissue-specific with a range of 3.43-4.26% of cytosine residues methylated in normal tissues (15,23,24). We used the *Cac*8I COBRA-DHPLC method to detect the methylated-*Alu *proportion in 48 pairs of GCs and GC-Nor and 55 gastric mucosa biopsy samples from noncancerous patients. Results showed that the average methylated-*Alu *proportion in GCs (%, mean ± SD, 3.01 ± 0.25) was significantly lower than that in GC-Nor (3.19 ± 0.31) (Figure 5; *P *< 0.01) and that in gastric biopsies from patients without tumor (3.23 ± 0.57; *P *< 0.001; data not shown). We did not observed any significant association between the methylated-*Alu *proportion in GCs and patients' clinical-pathological characteristics, such as lymph node metastasis, age, and sex (data not shown). This result is consistent with the hypothesis that globe hypomethylation of the genome is an early event during carcinogenesis [25-28].

## Conclusions

Most *Alu *CpG sites are deaminated in the genome. 27% of *Alu *CpG sites represented in our amplification products. 87% of the remaining CpG sites are methylated. Based on the analysis of extensive bisulfite clone sequencings, a *Cac*8I COBRA-DHPLC assay was developed to quantify sensitively the methylated-*Alu *proportion. Hypomethylation of *Alu *elements was observed in gastric carcinomas with the assay.

## Competing interests

The authors declare that they have no competing interests.

## Authors' contributions

SX participated in the design and conducted cloning studies; ZL contributed to the COBRA-DHPLC assay development and gastric sample analysis; BZ carried out cell culture and treated cells with 5-aza-dC; JZ extracted genomic DNA samples from tissues; B-DZ collected the gastric biopsy samples; JJ collected gastric carcinoma samples; DD designed and coordinated the study, analysed data, and wrote the manuscript. All authors read and approved the final manuscript.

## Pre-publication history

The pre-publication history for this paper can be accessed here:

http://www.biomedcentral.com/1471-2407/10/44/prepub

## Supplementary Material

Additional file 1 **Table S1. - Correlation coefficients of methylation status between each CpG site and *Alu *clone subgroups with various methylated CpGs**. All of 427 *Alu *clones were classified into different subgroups according to the methylated-CpG number. Average methylation frequency of each CpG site within each *Alu *subgroup was calculated (methylated-CpG number of the CpG site to the clone number of *Alu *within the subgroup). The correlation coefficient was calculated for each CpG site based on the corresponding average methylation frequency within each subgroup and the total methylated-CpG number within the subgroup. Click here for file
